# *Tropheryma whipplei* in Children with Gastroenteritis

**DOI:** 10.3201/eid1605.091801

**Published:** 2010-05

**Authors:** Didier Raoult, Florence Fenollar, Jean-Marc Rolain, Philippe Minodier, Emmanuelle Bosdure, Wenjun Li, Jean-Marc Garnier, Hervé Richet

**Affiliations:** Université de la Méditerranée, Marseille, France (D. Raoult, F. Fenollar, J.-M. Rolain, W. Li, H. Richet); Pôle de Maladies Infectieuses, Marseille (D. Raoult, F. Fenollar, J.-M. Rolain, H. Richet); Hôpital Nord, Marseille (P. Minodier. J.-M Garnier); Hôpital de la Timone, Marseille (E. Bosdure)

**Keywords:** Tropheryma whipplei, Whipple disease, diarrhea, gastroenteritis, bacteria, enteric infections, children, CME, research

## Abstract

*Tropheryma whipplei*, which causes Whipple disease, is found in human feces and may cause gastroenteritis. To show that *T*. *whipplei* causes gastroenteritis, PCRs for *T. whipplei* were conducted with feces from children 2–4 years of age. Western blotting was performed for samples from children with diarrhea who had positive or negative results for *T. whipplei*. *T. whipplei* was found in samples from 36 (15%) of 241 children with gastroenteritis and associated with other diarrheal pathogens in 13 (33%) of 36. No positive specimen was detected for controls of the same age (0/47; p = 0.008). Bacterial loads in case-patients were as high as those in patients with Whipple disease and significantly higher than those in adult asymptomatic carriers (p = 0.002). High incidence in patients and evidence of clonal circulation suggests that some cases of gastroenteritis are caused or exacerbated by *T. whipplei*, which may be co-transmitted with other intestinal pathogens.

## Abstract 2

This bacterium may be an etiologic agent of gastroenteritis.

## CME ACTIVITY

Medscape, LLC is pleased to provide online continuing medical education (CME) for this journal article, allowing clinicians the opportunity to earn CME credit. This activity has been planned and implemented in accordance with the Essential Areas and policies of the Accreditation Council for Continuing Medical Education through the joint sponsorship of Medscape, LLC and Emerging Infectious Diseases. Medscape, LLC is accredited by the ACCME to provide continuing medical education for physicians. Medscape, LLC designates this educational activity for a maximum of 0.5 *AMA PRA Category 1 Credits*™. Physicians should only claim credit commensurate with the extent of their participation in the activity. All other clinicians completing this activity will be issued a certificate of participation. To participate in this journal CME activity: (1) review the learning objectives and author disclosures; (2) study the education content; (3) take the post-test and/or complete the evaluation at **www.medscapecme.com/journal/eid**; (4) view/print certificate.

## Learning Objectives

Upon completion of this activity, participants will be able to:

- Describe the epidemiology of *Tropheryma whipplei* gastroenteritis
- Identify the bacterial load associated with *T. whipplei* gastroenteritis
- Specify laboratory findings associated with *T. whipplei* gastroenteritis
- Compare clinical findings of *T. whipplei* gastroenteritis with other infectious gastroenteritis.

## Editor

**Thomas J. Gryczan**, Copyeditor, *Emerging Infectious Diseases. Disclosure: Thomas J. Gryczan has disclosed no relevant financial relationships.*

## CME AUTHOR

**Charles P. Vega, MD**, Associate Professor; Residency Director, Department of Family Medicine, University of California, Irvine. *Disclosure: Charles P. Vega, MD, has disclosed no relevant financial relationships.*

## AUTHORS

Disclosures: **Didier Raoult, MD, PhD; Florence Fenollar, MD, PhD; Jean-Marc Rolain, PharmD, PhD; Philippe Minodier, MD; Emmanuelle Bosdure, MD; Wenjun Li, MD, PhD; Jean-Marc Garnier, PhD**; and **Hervé Richet, MD, PhD**, have disclosed no relevant financial relationships.

## Earning CME Credit

To obtain credit, you should first read the journal article. After reading the article, you should be able to answer the following, related, multiple-choice questions. To complete the questions and earn continuing medical education (CME) credit, please go to **www.medscapecme.com/journal/eid**. Credit cannot be obtained for tests completed on paper, although you may use the worksheet below to keep a record of your answers. You must be a registered user on Medscape.com. If you are not registered on Medscape.com, please click on the New Users: Free Registration link on the left hand side of the website to register. Only one answer is correct for each question. Once you successfully answer all post-test questions you will be able to view and/or print your certificate. For questions regarding the content of this activity, contact the accredited provider, CME@medscape.net. For technical assistance, contact CME@webmd.net. American Medical Association’s Physician’s Recognition Award (AMA PRA) credits are accepted in the US as evidence of participation in CME activities. For further information on this award, please refer to http://www.ama-assn.org/ama/pub/category/2922.html. The AMA has determined that physicians not licensed in the US who participate in this CME activity are eligible for *AMA PRA Category 1 Credits*™. Through agreements that the AMA has made with agencies in some countries, AMA PRA credit is acceptable as evidence of participation in CME activities. If you are not licensed in the US and want to obtain an AMA PRA CME credit, please complete the questions online, print the certificate and present it to your national medical association.

### Article Title: *Tropheryma whipplei* in Children with Gastroenteritis

## CME Questions

1. Which of the following statements about the epidemiology of *T****ropheryma*** *whipplei* gastroenteritis in the study cohort is most accurate?
  A. 15% of children with gastroenteritis have positive testing for *T. whipplei*
  B. The infection rate with *T. whipplei* increased gradually from 2006 to 2008
  C. Infection with *T. whipplei* was most common during the winter months
  D. There were significant rates of positive *T. whipplei* testing in children without diarrhea
2. Which of the following statements about the bacterial loads of children with positive testing for *T. whipplei* is most accurate?
  A. Bacterial loads were undetectable or low in the majority of children
  B. Bacterial loads were lower than that of chronic carriers
  C. Bacterial loads were comparable to those of individuals with Whipple's disease
  D. Bacterial loads generally remained elevated after the resolution of diarrhea
3. Which of the following statements about laboratory test results in children with *T. whipplei* gastroenteritis is most accurate?
  A. One genotype of *T. whipplei* was associated with all cases
  B. Co-infection with other pathogens was more common in patients with *T. whipplei* gastroenteritis compared with other children with diarrhea
  C. There was no difference in the rate of seropositivity for *T. whipplei* in comparing cases and controls
  D. Co-infection with other pathogens was limited to children with higher bacterial loads of *T. whipplei*
4. The following are clinical features of *T. whipplei* gastroenteritis compared with other infectious diarrhea, *except*:
  A. Shorter duration of hospitalization
  B. Shorter duration of fever
  C. Less anorexia
  D. Smaller degrees of weight loss

### Activity Evaluation

**Table:** 

**1. The activity supported the learning objectives.**				
Strongly Disagree				Strongly Agree
1	2	3	4	5
**2. The material was organized clearly for learning to occur.**				
Strongly Disagree				Strongly Agree
1	2	3	4	5
**3. The content learned from this activity will impact my practice.**				
Strongly Disagree				Strongly Agree
1	2	3	4	5
**4. The activity was presented objectively and free of commercial bias.**				
Strongly Disagree				Strongly Agree
1	2	3	4	5

## *Tropheryma whipplei* in Children with Gastroenteritis

For decades, Whipple disease was considered to be a metabolic disorder in humans (*1*). An accumulation of data, such as antimicrobial drug susceptibilities and observation of atypical bacteria in intestinal macrophages, has suggested that this disease is an infectious disease. *Tropheryma whipplei* is recognized as the infectious agent responsible for Whipple disease (*2*). Recent studies using molecular biology (*3**–**5*) and culture-dependent (*6**–**10*) techniques have enabled the *T. whipplei* genome to be fully sequenced (*11**,**12*) and have resulted in development of new culture media (*13*), selection of highly sensitive primers for quantitative PCR (*14*), and genotyping (*15**–**19*). The bacterium is found in a viable form in stools of infected patients (*9*).

Until recently, *T. whipplei* was considered a rare bacterium that caused an uncommon disease (*2*). However, recent studies have confirmed that *T. whipplei* is common in stool samples (*20**,**21*). *T. whipplei* DNA has been detected in sewage and is highly prevalent in the feces of sewer workers (12%–26%) (*1**,**20**,**22**–**25*). Moreover, the prevalence of *T. whipplei* in feces of healthy children 2–10 years of age who lived in rural Senegal (sub-Saharan Africa) was 44% (46/105) (*21*). These data, together with the genetic heterogeneity of *T. whipplei* (*19*), indicate that *T. whipplei* is a rather common gut bacterium.

We hypothesize that *T. whipplei* may cause gastroenteritis in children as a result of its primary contact with humans. In a preliminary study, we tested all patients with diarrhea at the University Hospitals in Marseille, France, and determined that this bacterium was found most often in children 2–4 years of age (*26*).

To investigate whether *T. whipplei* caused gastroenteritis, we studied the prevalence of *T. whipplei* DNA in a prospective study of children with diarrhea and controls with diarrhea. We genotyped *T. whipplei* to identify circulating clones (*9*) and report overall findings, including data from the preliminary study.

## Materials and Methods

### Patients

The study was reviewed and approved by the local ethics committee (agreement no. 07–006). A case-patient was defined as a child 2–4 years of age who had 2 positive quantitative PCR results specific for 2 *T. whipplei* DNA sequences, as reported (*14**,**20*). From January 2006 through December 2008, we tested 241 stool samples from 241 children with diarrhea at 2 University Hospitals for children in Marseille, France (Timone and Nord Hospitals) using a *T. whipplei*–specific PCR. Samples were obtained from all children in accordance with routine hospital procedures. All stools samples were handled identically. Among 36 children with gastroenteritis and *T. whipplei* DNA in stools, 11 stool specimens from 10 children were obtained after their recovery from diarrheal illness.

Five stool specimens were tested ≈15 days later and 6 were sampled ≈1 month later. Eight serum samples from children with gastroenteritis and positive PCR results for *T. whipplei* were tested retrospectively for *T*. *whipplei* by Western blot analysis. Data for patients with a definite diagnosis of Whipple disease and positive PCR results for *T*. *whipplei* PCR in stools at the time of diagnosis and data for adult asymptomatic carriers of *T. whipplei* in stools, which have been reported (*14**,**20*), were also included for quantitative comparisons.

### Controls

All *T. whipplei*–infected case-patients were compared with 67 gastroenteritis case-patients of the same age. These 67 children had negative results for *T. whipplei* in stool specimens; epidemiologic, clinical, and biologic features were available for these children.

Forty-seven stool specimens from children 2–4 years of age without gastroenteritis were also tested for *T*. *whipplei* by using PCR. Twenty-five children (11 girls and 14 boys) were from kindergarten classrooms at the university hospitals. Samples were also obtained from 10 children (4 boys and 6 girls) hospitalized at Timone Hospital for surgery and from 12 children (5 boys and 7 girls) who visited the Emergency Department of Nord Hospital.

Twenty-five serum samples obtained from children 2–4 year of age with gastroenteritis and *T. whipplei*–negative results in stool specimens were tested retrospectively. Twenty control serum samples were obtained from 20 children 1–36 months of age with a disease other than gastroenteritis. All serum samples were from children hospitalized in the 2 University Hospitals. These samples were obtained for routine management of these patients, not specifically for our study.

### Diagnostic Procedures

Bacteria, viruses, and *Giardia duodenalis* were detected by using standard methods. Stool specimens were plated onto Hektoen, Campylosel, and *Yersinia* cefsulodin–irgasan–novobiocin agar plates (bioMérieux, Marcy L’Etoile, France). Plates selective for *Campylobacter* spp. were incubated under microaerophilic conditions; all other media and samples were incubated in ambient air. Temperature of incubation was 37°C, with the exception of *Yersinia* agar, which was incubated at 30°C. Length of incubation was 5 days. For virus tests, stool specimens were tested by using a chromatographic immunoassay with a VIKIA Rota-Adeno Kit (bioMérieux) kit and electron microscopy with negative staining, which enabled detection of rotavirus, adenovirus, calicivirus, astrovirus, Norwalk virus, coronavirus, and enterovirus. *G*. *duodenalis* was detected by PCR, as described (*27*).

*T*. *whipplei* quantitative PCR assays of stool samples were performed as reported (*14**,**20**,**28*). Approximately 1 g of stool was obtained for DNA extraction by using the QIAamp DNA MiniKit (QIAGEN, Hilden, Germany), which was performed according to the manufacturer’s recommendations. The first *T. whipplei*–specific quantitative PCR specific for a 155-bp sequence used primer pair TW27 forward (5′-TGTTTTGTACTGCTTGTAACAGGATCT-3′) and TW182 reverse (5′-TCCTGCTCTATCCCTCCTATCATC-3′) and a Taqman probe (27 forward–182 reverse, 5′-6-FAM-AGAGATACATTTGTGTTAGTTGTTACA-TAMRA-3′). The PCR was conducted in a LightCycler (Roche Diagnostics, Meylan, France) (*14**,**20**,**28*) in a final volume of 20 µL that contained 10 µL of the probe, master kit (QIAGEN), 0.5 µL (10 pmol/µL) of each primer, 5 µL (2 µmol/µL) of probe, 2 µL of distilled water, and 2 µL of extracted DNA. The amplification conditions involved an initial denaturation step at 95°C for 15 min, followed by 40 cycles of denaturation at 95°C for 15 s, and annealing and elongation at 60°C for 60 s, with fluorescence acquisition in single mode. After every 5 samples, *T. whipplei*–negative controls (water, mixture, and human samples) were evaluated.

If the result of the first PCR was positive, it was systematically confirmed by a second PCR with a second set of primer pairs: TW13 forward (5′-TGAGTGATGGTAGTCTGAGAGATATGT-3′) and TW163 reverse (5′-TCCATAACAAAGACAACAACCAATC-3′). This second PCR used a Taqman probe (13 forward–163 reverse, 5′-6-FAM-AGAAGAAGATGTTACGGGTTG-TAMRA-3′) specific for a different 150-bp sequence, as described elsewhere; the same amplification conditions described above were used. For quantitative PCR, sequence-specific standard curves were generated by using 10-fold serial dilutions of a standard concentration of 10^6^ microorganisms of the Marseille-Twist *T. whipplei* strain. The number of transcript copies in each sample was then calculated from the standard curve by using LightCycler software.

Genotyping of *T. whipplei* from stool specimens was performed as reported (*19*). This analysis was specific for 4 highly variable genomic sequences (HVGS) and used primers TWT133 forward and reverse for HVGS 1, primers ProS forward and reverse for HVGS 2, primers SECA forward and reverse for HVGS 3, and primers TWT183 forward and reverse for HVGS 4. PCR was performed in a PTC-200 automated thermal cycler (MJ Research, Waltham, MA, USA), as reported (*19*).

Serologic assays were performed by using Western blotting as described (*29*). Before blotting, protein concentration was determined by using a commercial reagent (Bio-Rad, Hercules, CA, USA). *T. whipplei* Twist proteins were resuspended in Laemmli buffer (Sigma-Aldrich Chimie, Saint Quentin Fallavier, France) containing 100 mmol/L dithiothreitol to obtain a final protein concentration of 0.5 µg/µL. The protein lysate was heated for 5 min at 100°C. Five micrograms of native protein was loaded into wells of a 7.5% polyacrylamide gel, and proteins were resolved by sodium dodecyl sufate–polyacrylamide gel electrophoresis.

Proteins were then transferred to nitrocellulose membranes (Transblot Transfer Medium, Pure Nitrocellulose Membrane, 0.45 mm; Bio-Rad) over a 2-hour period by using a semidry transfer unit (Hoeffer TE 77; GE Healthcare, Little Chalfont, UK). Membranes were immersed in phosphate-buffered saline supplemented with 0.2% Tween 20 and 5% non-fat dry milk (blocking buffer) for 1 h at room temperature and incubated with primary serum (dilution 1:1,000 in blocking buffer) for 1 h at room temperature. Membranes were then washed in triplicate with phosphate-buffered saline–Tween 20, and immunoreactive spots were detected by incubating the membranes for 1 h at room temperature with peroxidase-conjugated goat anti-human antibody (Southern Biotech, Birmingham, AL, USA) diluted 1:1,000 in blocking buffer. The first screening was performed by testing for all immunoglobulins (Igs). Positive cases were then tested to separately detect IgG and IgM. Detection was performed by using chemiluminescence (Enhanced Chemiluminescence Western Blotting Analysis System; Amersham Biosciences, Uppsala, Sweden) with an automated film processor (Hyperprocessor; GE Healthcare).

### Statistical Analysis

Data were analyzed by using EpiInfo software version 3.4.1 (Centers for Disease Control and Prevention, Atlanta, GA, USA). Proportions were compared by using the Yates χ^2^ corrected test or Fisher exact test. Continuous variables were compared by using analysis of variance or the Mann-Whitney/Wilcoxon 2-sample test when data were not normally distributed. Significance was defined as p<0.05.

## Results

A total of 241 children 2–4 years of age with gastroenteritis were tested, and samples from 36 (15%) were positive for *T*. *whipplei*. In 2006, 2007, and 2008, the infection rates for *T. whipplei* were comparable: 12/78 (15.4%), 10/72 (14%), and 14/91 (15.4%), respectively. No seasonal variation was observed. None of the children in the same age control group without diarrhea had samples positive for *T*. *whipplei* (0/47; p = 0.008). High bacterial loads (>10^4^/g of stool) were observed in 64% (23/36) of the *T*. *whipplei*–positive children. Such high loads have not been observed in chronic carriers, but they were comparable to the levels for patients with Whipple disease (*14*). Bacterial load in stools ranged from 170 to 1.5 × 10^6^/g (mean ± SD 1.5 × 10^5^ ± 3.6 × 10^5^) for children with gastroenteritis versus 85 to 2.5 × 10^6^/g (mean ± SD 5.5 × 10^5^ ± 8.3 × 10^5^) for patients with Whipple disease (p = 0.1). Only 1 postdiarrheal stool specimen was slightly positive 15 days later and had a bacterial load <85/g of stool; the bacterial load was 2 × 10^4^ at the time of diarrhea. Stool samples obtained from the same patient 1 month later were negative by PCR.

**Figure 1:**
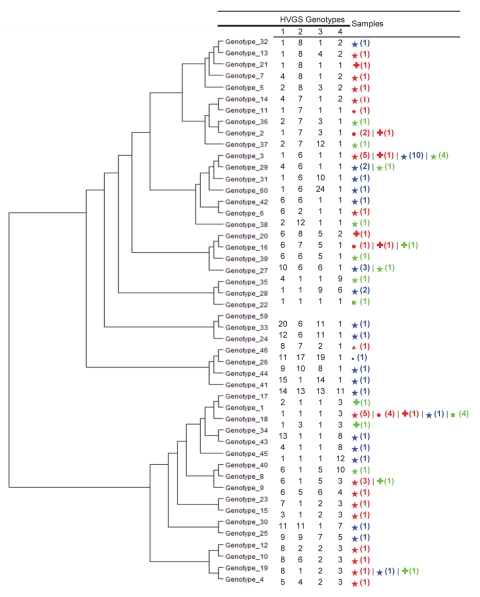
Dendogram constructed by using unweighted pair group method with arithmetic mean and 4 highly variable genomic sequences (HVGS), showing phylogenetic diversity of 48 genotypes of 81 *Tropheryma whipplei* strains detected in 34 children with diarrhea (blue), 40 adult patients with Whipple disease (red), and 22 asymptomatic adult patients without Whipple disease (green) (including 11 sewer workers), Marseille, France. Sequences were concatenated to construct the dendrogram. Numbers in parentheses indicate number of genotyped samples for each category. Stars, France; crosses, Switzerland; circles, Germany; diamond, Italy; square, Austria; triangle, Canada; small circle, Comoros.

**Figure 2:**
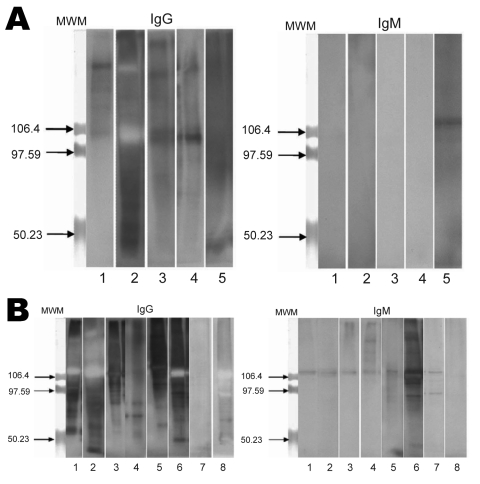
Western blot analysis of immunoglobulin (Ig) G and IgM against *Tropheryma whipplei* for children with gastroenteritis, Marseille, France. Total native antigens from *T. whipplei* were tested. A) Five patients without *T. whipplei* detected from stool samples but with positive Western blot serologic results. B) Eight patients infected with *T. whipplei*. MWM, molecular weight markers. Values on the right of each blot are in kilodaltons.

Genotyping of *T. whipplei* was performed for 34 children with diarrhea. A dendogram showing phylogenetic organization of genotypes is shown in Figure 1. We observed genetic heterogeneity in sequences associated with gastroenteritis and identified 12 new genotypes. One useful finding was that genotype 3 was detected in 10 of the 34 children. Using Western blotting, we found that case-patients with Whipple disease were significantly more likely to be seropositive than controls with diarrhea (Figure 2) for IgG (7/8 vs. 5/25; p = 0.001) and IgM (7/8 vs. 1/25; p<0.001) and than controls without gastroenteritis for IgG (5/20; p = 0.004) and IgM (1/20; p<0.001).

Clinical and biological features of the 36 PCR-positive children 2–4 years of age with diarrhea were compared by retrospective review with those of matched 67 control children of the same age who had gastroenteritis but were negative for *T. whipplei* by PCR. Results are summarized in Table 1. Among children with positive PCR results, the proportion of girls and boys was equal. *T*. *whipplei* PCR-positive patients with diarrhea had a milder illness than PCR-negative patients. Length of rehydration required, duration of fever, and duration of hospitalization were significantly shorter for the patients infected with *T*. *whipplei* (p = 0.003, p = 0.003, and p = 0.01, respectively). Moreover, the level of C-reactive protein was significantly lower incase-patients (p = 0.03). Patients with *T*. *whipplei* were less likely to experience anorexia (p = 0.03); however, their weight loss was significant (p = 0.045). Another significant difference was increased contact with sand boxes for case-patients (p = 0.002); however, the size of the group analyzed was small, and these data should be confirmed.

**Table 1:** Clinical and biological characteristics and laboratory test results of children 2–­4 years of age with gastroenteritis who tested positive by PCR for *Tropheryma whipplei*, compared with controls, Marseille, France*

Characteristic	*T. whipplei* PCR positive, n = 36	*T. whipplei* PCR negative, n = 67†	p value
Male sex	19 (53)	35 (52)	0.9
Median age, mo	29 ± 6	29 ± 5	0.7
Sand box contact	4/4 (100)	0/8 (0)	0.002
Anorexia	14 (39)	41 (62)	0.03
Weight loss, kg	0.9 ± 0.6	0.55 ± 0.35	0.045
Vomiting	20 (55.5)	45 (67)	0.2
Abdominal pain	9 (25)	14 (21)	0.3
Dehydration	11 (30.5)	25 (37)	0.5
Watery diarrhea	25 (69)	46 (69)	0.9
Bloody diarrhea	3 (8)	5 (7.5)	0.9
Duration of diarrhea, d	4.5 ± 4	4.5 ± 4	0.9
No. stools/d	6 ± 3	5.5 ± 3	0.2
Length of rehydration required, h	19 ± 10	35 ± 23	0.003
Fever, °C	38 ± 0.9	38.3 ± 1	0.2
Duration of fever, d	1.4 ± 1	3.6 ± 3.7	0.003
Duration of hospitalization, d	1.6 ± 1.5	3.5 ± 3.8	0.01
Peripheral leukocyte count, × 10^9^/L	10 ± 4	14 ± 7	0.2
Neutrophil count, × 10^9^/L	6 ± 3	7.5 ± 6	0.7
Hemoglobin, g/dL	11.8 ± 1.5	11.9 ± 1.5	0.6
Fibrin, g/L	4 ± 0.8	4.2 ± 1.2	0.6
C-reactive protein, mg/mL	24 ± 23	64 ± 87	0.03
Serum albumin, g/L	37 ± 50.2	34 ± 3	0.2
Serum creatinine, mmol/L	37 ± 11	37 ± 12	0.99
Uremia, mmol/L	3.4 ± 2	3.4 ± 2	0.99
Proteinemia, g/L	69 ± 8	68.5 ± 7	0.7
Serum potassium, mmol/L	3.8 ± 0.5	3.9 ± 0.4	0.3
Partial thromboplastin time, s	31 ± 3	32 ± 5	0.6
Prothrombin time, s	83 ± 20	81 ± 17	0.7

*Values are no. (%), no. positive/no. tested (%), or mean ± SD. †Among 205 case-patients with gastroenteritis who were negative for *T. whipplei*, data were available for 67 patients.

Children infected with *T*. *whipplei* were co-infected with an associated pathogen more often than patients with diarrhea without *T. whipplei* infection (13/36 vs. 36/205; p = 0.01) (Table 2). Co-infection was less common in 23 children with high *T. whipplei* bacterial loads (>10^4^/g of stool) than in 13 children with lower bacterial loads (5/23 vs. 8/13; p = 0.02).

**Table 2:** Microbiologic data for stool specimens of 241 children, Marseille, France*

Pathogen	*T. whipplei*–positive children			*T. whipplei*–negative children
	Bacterial load <10^4^/g of stool	Bacterial load >10^4^/g of stool	All	
Any other	8/13	5/23	13/36	36/205
Bacteria				
*Salmonella* sp.	1 (1 with *Giardia duodenalis*)	2	3 (1 with *G. duodenalis*)	2
Other	1 with *Campylobacter jejuni* (also associated with rotavirus)	0	1 with *C. jejuni* (also associated with rotavirus)	5 (1 *C. jejuni,* 1 *Escherichia coli* O26:B6, 1 *E. coli* O126:B16 with 1 rotavirus, 1 *Shigella sonnei,* and 1 *Yersinia enterocolitica*)
Viruses				
Rotavirus	5	3	8 (1 also associated with *C. jejuni*)	21
Other	2 (1 adenovirus and 1 calicivirus)	0	2 (1 adenovirus and 1 calicivirus)	9 (4 enterovirus, 3 adenovirus, and 2 calicivirus)

*Testing for *Tropheryma* whipplei was conducted by using PCR.

## Discussion

*T*. *whipplei* has been identified by PCR in stools of persons without Whipple disease (*20*). The source of *T*. *whipplei* is unknown, but data suggest that it the infection may result from fecal–oral or oral–oral transmission (*2**,**20*). *T*. *whipplei* is excreted in a live form by patients with Whipple disease (*9*), and *T*. *whipplei* DNA is found in stool samples of healthy persons (*22*). This bacterium is commonly found in sewers (*20**,**23*) and has also been detected in sewage (*25*). Therefore, it appears that *T*. *whipplei* is much more common than previously believed. A total of 3.8% (7/299) of adult controls in our study were PCR positive for *T. whipplei* (*14*). The bacterial load of *T. whipplei* in stools is much lower in asymptomatic carriers than in patients with Whipple disease (*14**,**20*).

We believe that our data provide strong evidence to support our initial hypothesis that *T. whipplei* causes mild gastroenteritis in children 2–4 years of age. All of our case-patients had 2 quantitative PCR-positive results, and genotyping of *T. whipplei* from 32 children enabled us to identify 12 new genotypes. Our data exclude the possibility of PCR contamination. We also found bacterial loads higher than those in previous reports of chronic carriers (*20*). However, these loads were comparable with those for patients with active Whipple disease (*14*). These high loads suggest that gastroenteritis in the children in our study was associated with active *T. whipplei* replication.

The absence of *T*. *whipplei* DNA in stools after patient recovery from diarrheal illness strongly suggests that detection of *T*. *whipplei* DNA is associated with acute gastroenteritis rather than carriage, which is usually chronic (*20*). Moreover, 1 genotype (genotype 3) predominated, causing one third of the total number of cases.

Serologic analysis by Western blotting for *T. whipplei* (*29*) showed that all children who were PCR positive for *T*. *whipplei* had IgM against *T. whipplei*, which suggested that recent seroconversion had occurred. The prevalence of antibodies in case-patients was higher than that in controls. Comparison of the prevalence of antibodies for control children (22%) with preliminary prevalence data for persons 61 ± 3.6 years of age (45%) (*30*) showed that the difference is significant (p<0.001); thus, possible acquired immunity to infection with *T. whipplei* may explain why this bacterium causes diarrhea in children, but not in most adults.

We suspect that *T. whipplei* infection is contagious and transmitted by the fecal–oral route in children 2–4 years of age with other enteric pathogens. We previously reported an association between *G*. *duodenalis* infection and Whipple disease (*27*). As with other organisms such as *Helicobacter pylori*, *T. whipplei* may also be transmitted through saliva because it has been found in saliva of asymptomatic carriers and patients with Whipple disease (*20*). Therefore, we hypothesized that primary infection with *T. whipplei* in children may result in gastroenteritis, especially when associated with other intestinal pathogens. We have provided several lines of evidence that *T. whipplei* is causing or exacerbating gastroenteritis. The incidence of *T. whipplei* DNA in stools of children 2–4 years of age with gastroenteritis was higher than that in the control group; these infected children also have higher levels of antibodies. That healthy children of the same ages were not infected with *T. whipplei* also suggests that primary infection is symptomatic. Sociodemographic differences between the 2 groups could theoretically explain our findings. However, most of our patients and controls were from the same geographic area, which enabled us to rule out this hypothesis.

We identified 1 clone (genotype 3) in 10 children, which indicates that this clone is circulating in our population. The association we found between *T*. *whipplei* and other pathogens transmitted by the fecal–oral route supports the conclusion that *T*. *whipplei* and other intestinal pathogens have a common source of infection and that they are often co-transmitted (*31**,**32*). However, for unknown reasons, *T*. *whipplei* could replicate in children with low-grade chronic infections without causing diarrhea. That *T. whipplei*–infected patients were more likely to have co-infections than patients infected with other pathogens may also indicate that *T*. *whipplei* may decrease below molecular detection limits when diarrhea resolves. However, higher levels of IgM against *T*. *whipplei* in case-patients suggest infection with this bacterium. Moreover, an in vivo animal model of oral infection by *T*. *whipplei* has shown a pathogenic effect only in mice with previously inflamed colonic tissues (D. Raoult et al., unpub. data). Thus, inflamed colonic tissues may also explain the frequency of co-infections with common pathogens observed in persons with *T. whipplei*–positive gastroenteritis.

We provide evidence that *T*. *whipplei* is commonly associated with gastroenteritis in children. We also suggest that other studies should be performed to evaluate the role of this bacterium and its prevalence in patients with gastroenteritis because it is present worldwide.
